# Supplementary material for: Reprogrammed CRISPR-Cas13b suppresses SARS-CoV-2 replication and circumvents its mutational escape through mismatch tolerance
Source: Nat Commun. 2021 Jul 13;12:4270. doi: 10.1038/s41467-021-24577-9 (PMC8277810; doi:10.1038/s41467-021-24577-9)
Supplement: Supplementary file 1 — Supplementary Information [file 41467_2021_24577_MOESM1_ESM.pdf]

Supplementary Fig. 1

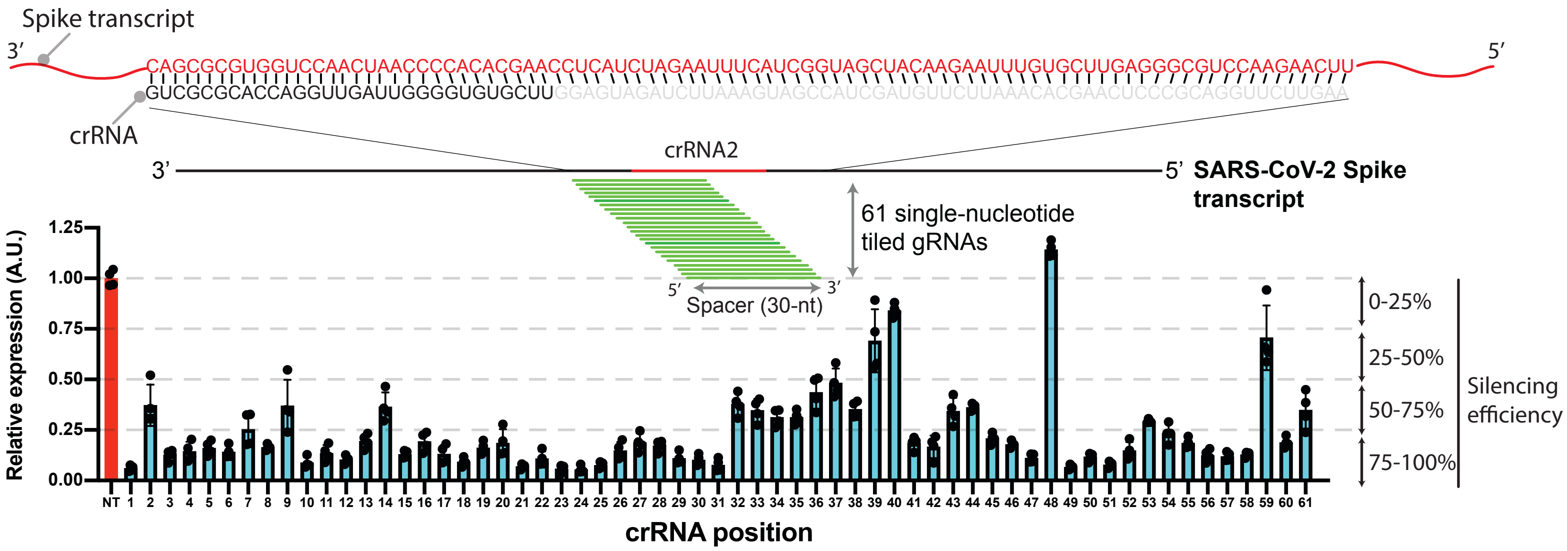

Supplementary Figure 1. Tiling of 61 crRNAs with single-nucleotide increment reveals that RNA sequence, position, and/or landscape influence pspCas13b silencing efficiency. The schematic shows the sequence of target RNA covered by 61 tiled crRNAs. Data are normalized mean fluorescence per field of view. Data points in the graph are normalized mean fluorescence from 4 representative fields of view imaged in N=1. N is the number of independent biological experiments. This is an independent biological replicate that reproduces the data in **Fig. 1G**. Source data are provided as a Source data file.

Supplementary Figure 2

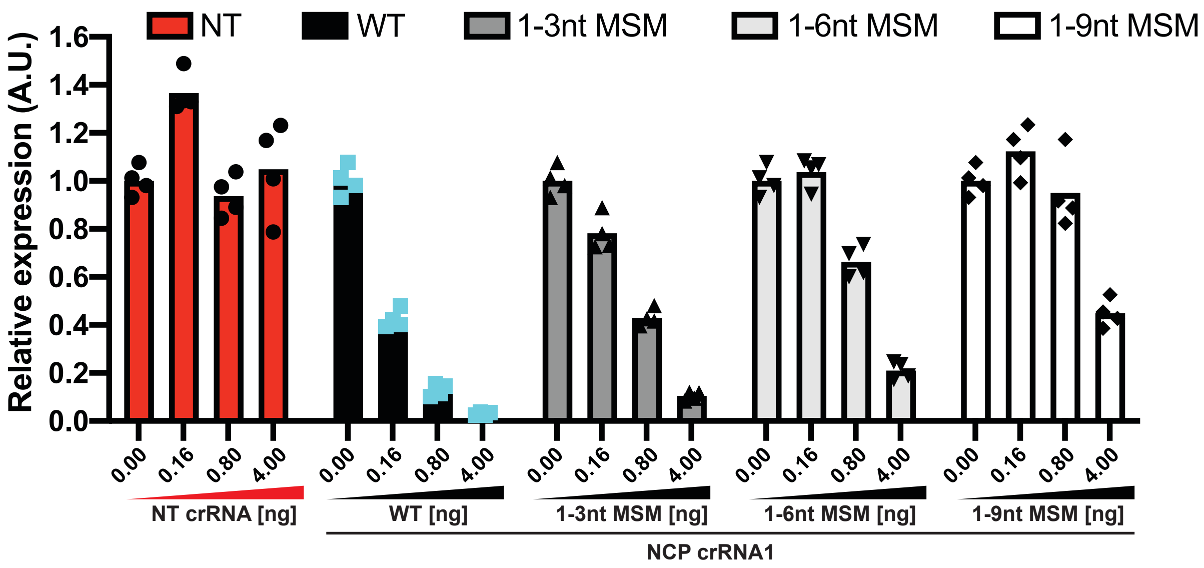

Supplementary Figure 2. Dose-dependent silencing of NCP transcript with non-targeting crRNA (NT), wildtype crRNA1 (WT), crRNA1 harbouring 1-3, 1-6, or 1-9 nucleotides mismatch at the 5'end of the spacer. Data points in the graph are normalized mean fluorescence from 4 different field of views imaged in N=1. N is the number of independent biological experiments. Source data are provided as a Source data file.

Note: The dose-dependent silencing shows that the silencing efficiency is dependent on the degree of base-pairing between the spacer and the target. The reduction in the silencing efficiency is proportional to the number of mismatches within the spacer-target RNA-RNA duplex. For instance, with 0.8ng of WT, 1-3, 1-6, and 1-9 nucleotides mismatch crRNAs we achieved 87%, 57%, 34%, and 5% silencing efficiency, respectively. Thus, demonstrating the dependency between the spacer-target base-pairing and silencing efficiency.

Supplementary Figure 3

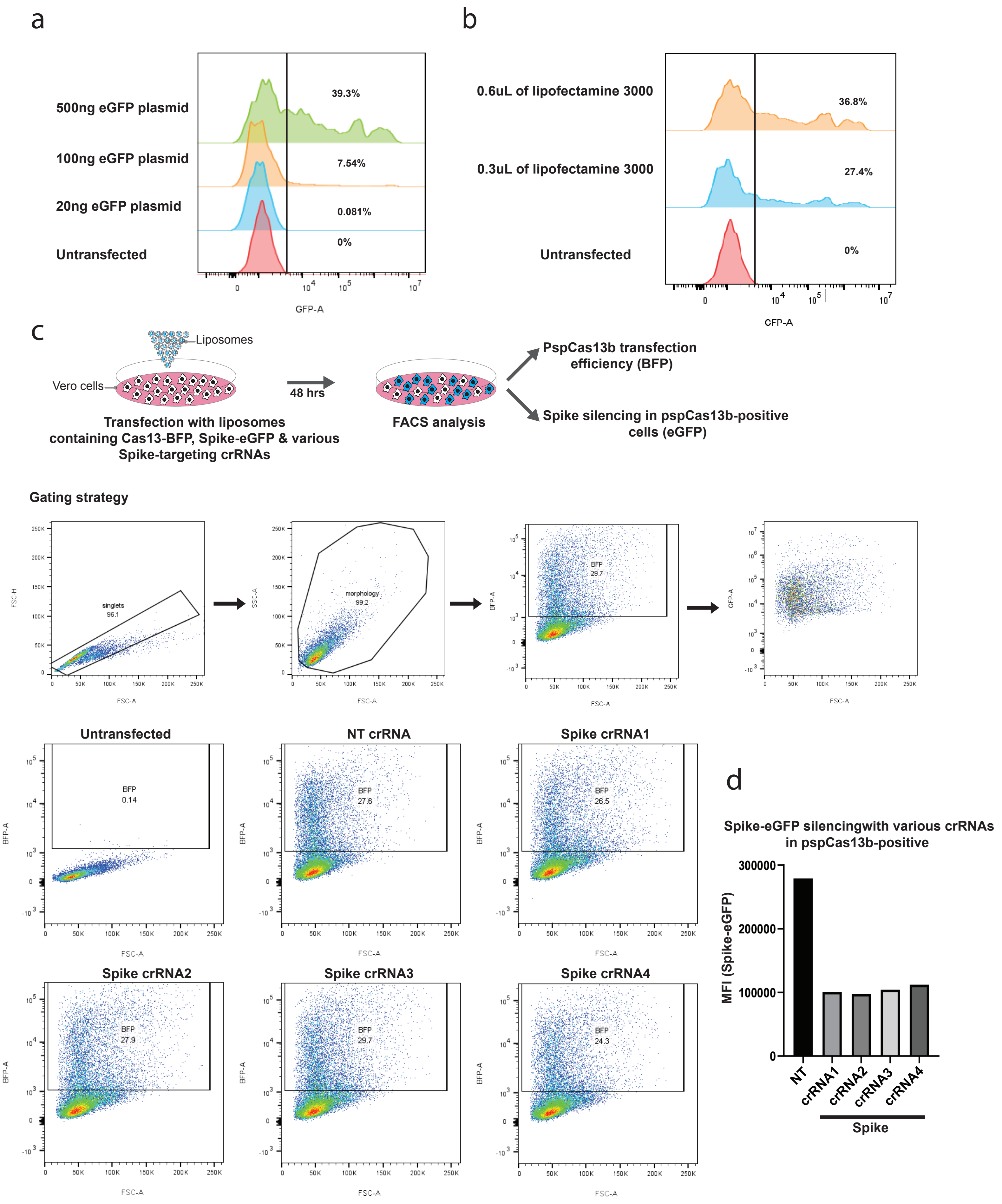

Supplementary Figure 3. Optimization of VERO cells transfection conditions.

(a) 20,000 VERO cells were seeded in a 96-well plate and transfected with various amounts of Spike-eGFP reporter plasmid (0-500ng). 48h later, the transfection efficiency was measured by flow cytometry (FACS). N=1. N is the number of independent biological experiments

(b) 20,000 VERO cells were seeded in a 96-well plate and transfected with 100ng of Spike-eGFP reporter plasmid packaged in either 0.3μL or 0.6μL of lipofectamine 3000. Flow cytometry analysis showed that 0.6μL of lipofectamine 3000 gave the highest transfection efficiency (36.8%). N=1. N is the number of independent biological experiments.

(c) FACS analysis of pspCas13b-BFP transfection efficiency in VERO cells. VERO cells were transfected with pspCas13b-BFP, Spike-eGFP, and either NT or four Spike-targeting crRNAs. Both transfection and silencing efficiencies were measured by FACS 48h post-transfection. N=1. N is the number of independent biological experiments.

The gating strategy is shown in the upper panels. In this experiment, 25-30% of VERO cells expressed detectable levels of pspCas13b-BFP.

(d) The histogram quantifies eGFP mean fluorescence intensity (MFI) in each transfection condition that reflects the silencing of Spike-eGFP RNA in pspCas13b-positive VERO cells; N=1. N is the number of independent biological replicates. Source data are provided as a Source data file.

Suppl. Figure 4

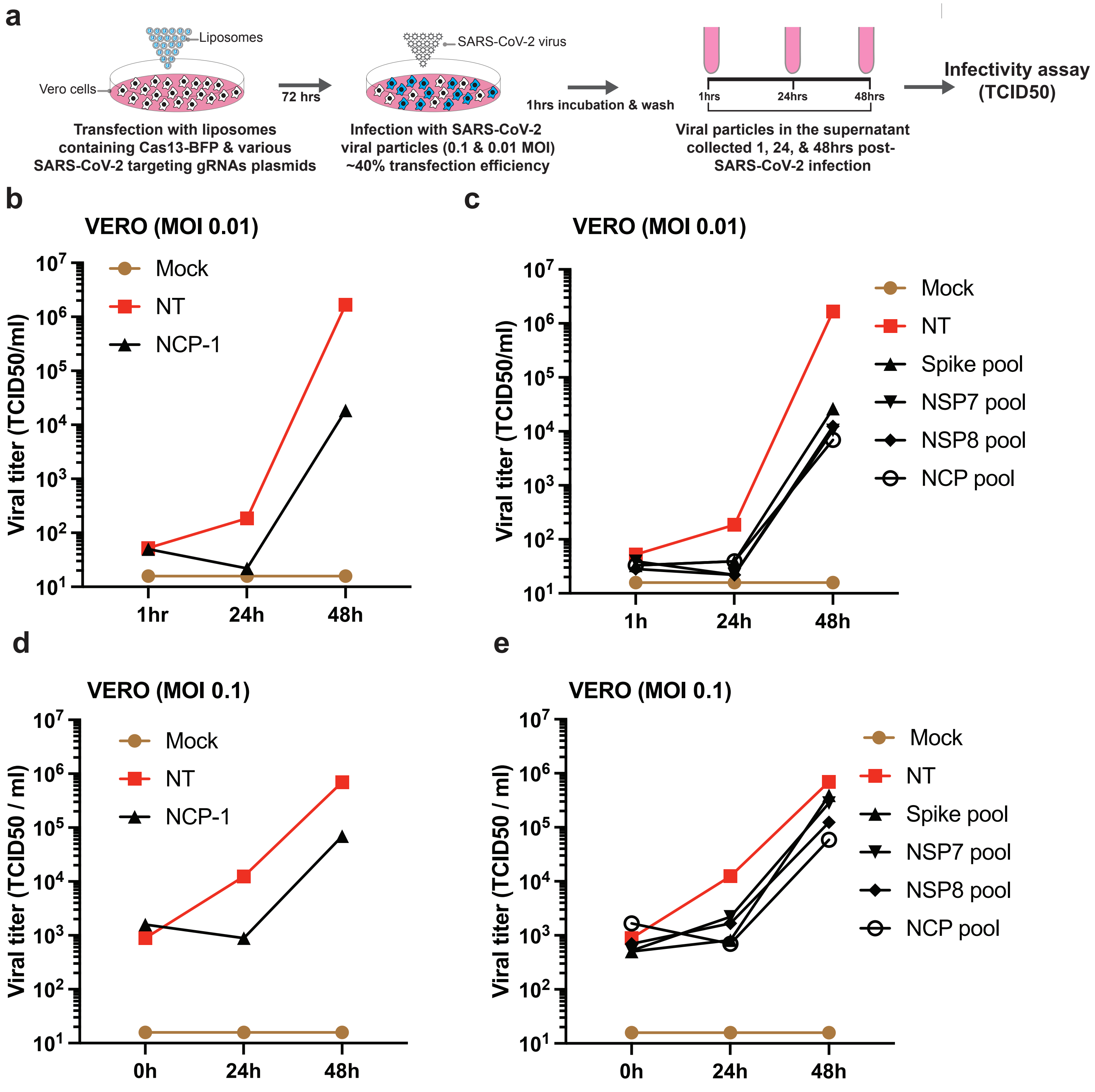

Supplementary Figure 4. Silencing of replication-competent SARS-CoV-2 virus in infected VERO cells. **(a)** Schematic of infection assay to assess pspCas13b-mediated suppression of SARS-CoV-2 replication in infected VERO cells. VERO cells were transfected with liposomes containing pspCas13b-BFP and various crRNA constructs. 72h post-transfection, cells were infected with SARS-CoV-2 for 1 hour and the kinetic of viral replication was measured by assessing the infectivity of virus shed into the culture medium at 1, 24, and 48hrs post-infection.
(b, d) infectivity assay to evaluate the kinetics of viral replication in VERO cells expressing either NT or NCP-targeting crRNA1 at 0.01 **(b)** or 0.1 MOI **(d)**.
(c, e) infectivity assays to monitor the kinetics of viral replication in VERO cells expressing either NT or various pools of crRNA targeting NCP, Spike, NSP7 (RdRP subunit 7), or NSP8 at 0.01 **(c)** or 0,1 **(e)** MOI. Data points in all graphs in this figure are obtained from N=2 independent biological experiments, except for Mock (no SARS-CoV-2 infection) where N=1. N is the number of independent biological replicates. Source data are provided as a Source data file.

Supplementary Figure 5

a

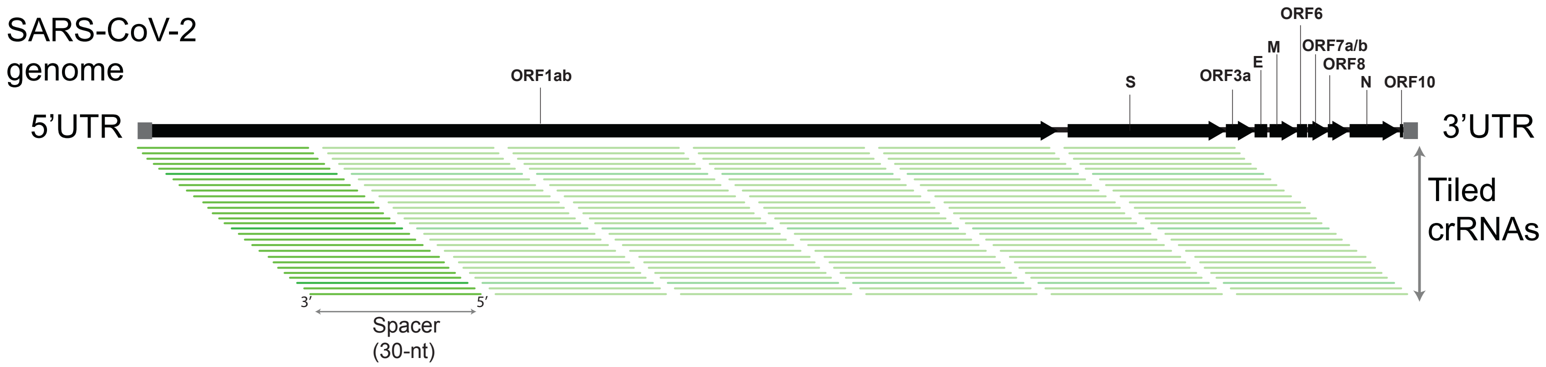

Single-nucleotide tiled crRNAs covering the entire genome of SARS-CoV-2 (Suppl. Table 1) (29,874 crRNAs)

Spacer containing ≥4T successive repeats are removed (Suppl. Table 2) (24,973 crRNAs)

Top crRNAs with predicted open secondary structure in the spacer and target sequence & lacking ≥4C repeats (Suppl. Table 3) (838 crRNAs)

Off-target prediction and removal of crRNA with 6 nucleotides or less mismatches with the whole human transcriptome (Suppl. Table 4) (494 crRNAs)

b

Top scoring crRNA

Spacer of crRNA10005-10035

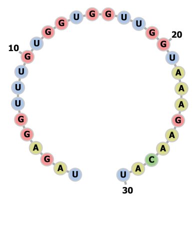

Target of crRNA10005-10035

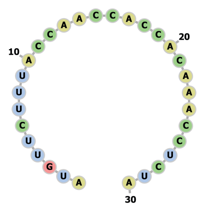

Lowest scoring crRNA

Spacer of crRNA 29024_29053

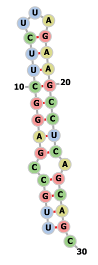

Target of crRNA 29024_29053

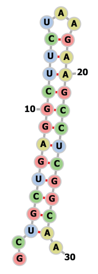

Supplementary Figure 5. (a) Bioinformatics workflow for the design and selection of pspCas13b crRNAs that are predicted to achieve high silencing efficiency. crRNAs are tiled across the entire RNA genome of SARS-CoV-2 with single-nucleotide increments. crRNAs harbouring 4 or more successive T/C residues are removed to avoid premature termination of Pol III-driven crRNA transcription, and the targeting of inaccessible G-quadruplex regions on the target transcript. The top 838 crRNAs with predicted open secondary structure in the spacer and target are selected (Supplementary Table 4). Based on our mutagenesis data (Figure 1 & 2), the prediction of off-targeting allowed the exclusion of 344 crRNAs that partially match the human transcriptome with six nucleotides or less mismatches. This conservative approach of off-targeting prediction allowed the selection of 494 crRNAs with minimum risk of off-targeting (Supplementary Table 4 & 5).
(b) Predicted secondary structure of the spacer (upper panels) and its target sequence (lower panels) in the top scoring and lowest scoring crRNAs in Suppl. Table 1. The RNA secondary structures were generated using the RNAfold program2 (ViennaRNA webservices). The top scoring crRNA shows a predicted open structure for both the spacer and the target, while both structures of the lowest scoring crRNA exhibit high probability of folding into stem-loop that may impair crRNA loading into pspCas13b and target accessibility.

Supplementary Figure 6

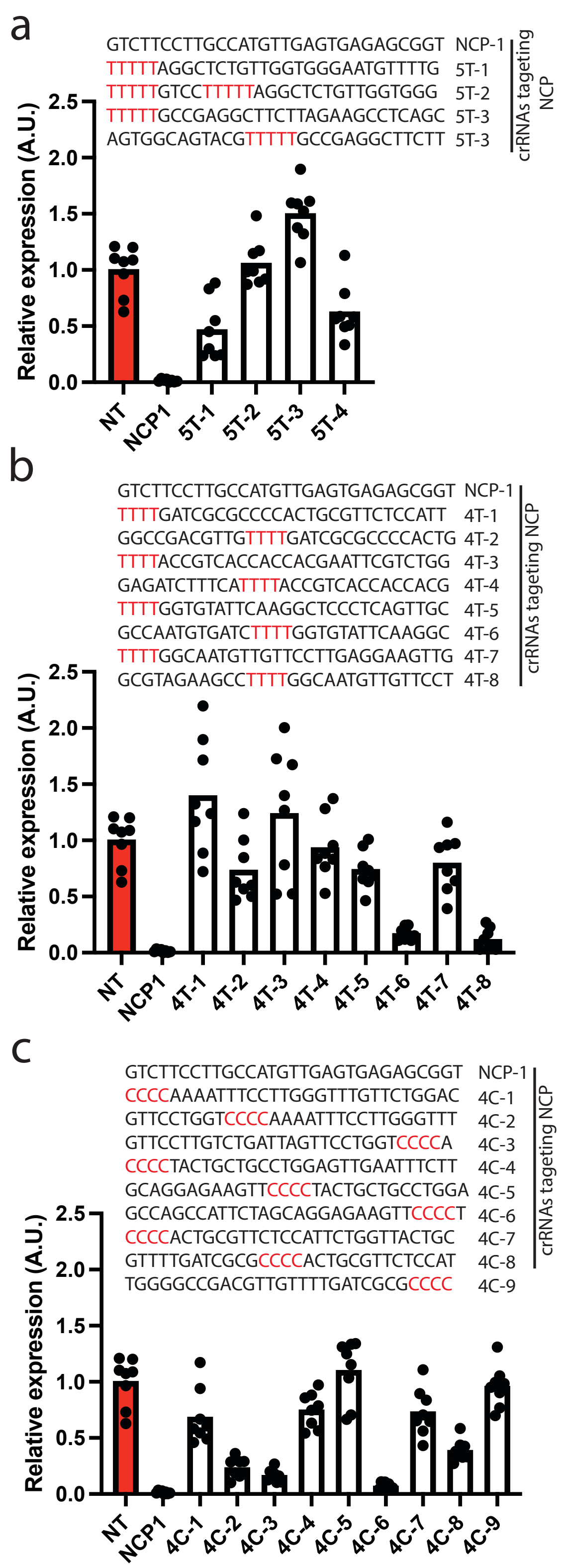

Supplementary Figure 6. (a-c) Probing the silencing efficiency of NCP transcript with crRNAs harbouring 5T, 4T, or 4C in their spacer sequence. Quantification of pspCas13b silencing efficiency with NCP-targeting crRNAs harbouring 5T **(a)**, 4T **(b)**, or 4C successive sequences **(c)** at various spacer locations in HEK 293T cells. 4 representative field of views were imaged per condition in N=2. N is the number of independent biological experiments. Source data are provided as a Source data file.

Supplementary Figure 7

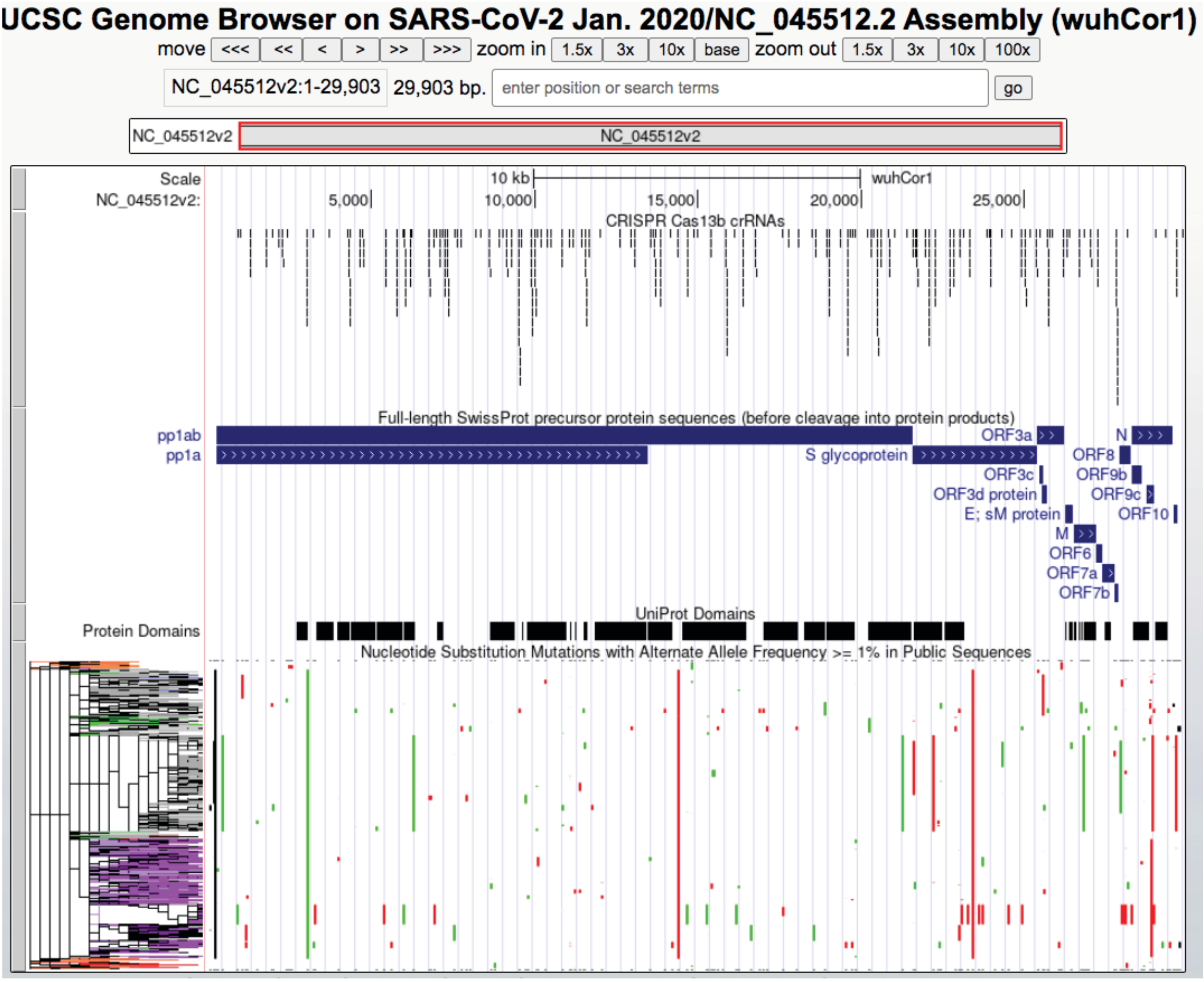

Supplementary Figure 7. Alignment of the top 838 crRNAs (Supplementary Table 3) to SARS-CoV-2 genome using UCSC genome browser. This tool allows for accurate mapping of crRNAs to the target SARS-CoV-2 genome and highlights in real-time emerging mutation hotspots and their overlap with crRNAs. The avoidance of mutation hotspots is a conservative approach to avoid viral escape in the unlikely event of several mutations occurring within a targeted sequence of a single crRNA (e.g. deletion or substitution of several bases.

Supplementary Figure 8

cDNA sequences of pspCas13b or SARS-CoV-2 constructs used in this study.

PspCas13b-3xFLAG-T2A-BFP.

a t g a a c a t c c c c g c t c t g g t g g a a a a c c a g a a g a a g t a c t t t g g c a c c t a c a g c g t g a t g g-
ccatgctgaacgctcagaccgtgctggaccacatccagaaggtggccgatattgagggcgagcagaacgagaacaacgagaatctgtggtt
tcaccccgtgatgagccacctgtacaacgccaagaacggctacgacaagcagcccgagaaaaccatgttcatcatcgagcggctgcagag
ctacttcccattcctgaagatcatggccgagaaccagagagagtacagcaacggcaagtacaagcagaaccgcgtggaagtgaacagca
acgacatcttcgaggtgctgaagcgcgccttcggcgtgctgaagatgtacagggacctgaccaaccactacaagacctacgaggaaaagct
gaacgacggctgcgagttcctgaccagcacagagcaacctctgagcggcatgatcaacaactactacacagtggccctgcggaacatgaa
cgagagatacggctacaagacagaggacctggccttcatccaggacaagcggttcaagttcgtgaaggacgcctacggcaagaaaaagtc
ccaagtgaataccggattcttcctgagcctgcaggactacaacggcgacacacagaagaagctgcacctgagcggagtgggaatcgccctg
ctgatctgcctgttcctggacaagcagtacatcaacatctttctgagcaggctgcccatcttctccagctacaatgcccagagcgaggaacggcg
gatcatcatcagatccttcggcatcaacagcatcaagctgcccaaggaccggatccacagcgagaagtccaacaagagcgtggccatggat
atgctcaacgaagtgaagcggtgccccgacgagctgttcacaacactgtctgccgagaagcagtcccggttcagaatcatcagcgacgacc
acaatgaagtgctgatgaagcggagcagcgacagattcgtgcctctgctgctgcagtatatcgattacggcaagctgttcgaccacatcaggtt
ccacgtgaacatgggcaagctgagatacctgctgaaggccgacaagacctgcatcgacggccagaccagagtcagagtgatcgagcagc
ccctgaacggcttcggcagactggaagaggccgagacaatgcggaagcaagagaacggcaccttcggcaacagcggcatccggatcag
agacttcgagaacatgaagcgggacgacgccaatcctgccaactatccctacatcgtggacacctacacacactacatcctggaaaacaac
aaggtcgagatgtttatcaacgacaaagaggacagcgccccactgctgcccgtgatcgaggatgatagatacgtggtcaagacaatcccca
gctgccggatgagcaccctggaaattccagccatggccttccacatgtttctgttcggcagcaagaaaaccgagaagctgatcgtggacgtgc
acaaccggtacaagagactgttccaggccatgcagaaagaagaagtgaccgccgagaatatcgccagcttcggaatcgccgagagcgac
ctgcctcagaagatcctggatctgatcagcggcaatgcccacggcaaggatgtggacgccttcatcagactgaccgtggacgacatgctgac
cgacaccgagcggagaatcaagagattcaaggacgaccggaagtccattcggagcgccgacaacaagatgggaaagagaggcttcaag
cagatctccacaggcaagctggccgacttcctggccaaggacatcgtgctgtttcagcccagcgtgaacgatggcgagaacaagatcaccg
gcctgaactaccggatcatgcagagcgccattgccgtgtacgatagcggcgacgattacgaggccaagcagcagttcaagctgatgttcgag
aaggcccggctgatcggcaagggcacaacagagcctcatccatttctgtacaaggtgttcgcccgcagcatccccgccaatgccgtcgagttc
tacgagcgctacctgatcgagcggaagttctacctgaccggcctgtccaacgagatcaagaaaggcaacagagtggatgtgcccttcatccg
gcgggaccagaacaagtggaaaacacccgccatgaagaccctgggcagaatctacagcgaggatctgcccgtggaactgcccagacag
atgttcgacaatgagatcaagtcccacctgaagtccctgccacagatggaaggcatcgacttcaacaatgccaacgtgacctatctgatcgcc
gagtacatgaagagagtgctggacgacgacttccagaccttctaccagtggaaccgcaactaccggtacatggacatgcttaagggcgagta
cgacagaaagggctccctgcagcactgcttcaccagcgtggaagagagagaaggcctctggaaagagcgggcctccagaacagagcggt
acagaaagcaggccagcaacaagatccgcagcaaccggcagatgagaaacgccagcagcgaagagatcgagacaatcctggataag
cggctgagcaacagccggaacgagtaccagaaaagcgagaaagtgatccggcgctacagagtgcaggatgccctgctgtttctgctggcca
aaaagaccctgaccgaactggccgatttcgacggcgagaggttcaaactgaaagaaatcatgcccgacgccgagaagggaatcctgagc
gagatcatgcccatgagcttcaccttcgagaaaggcggcaagaagtacaccatcaccagcgagggcatgaagctgaagaactacggcgac
ttctttgtgctggctagcgacaagaggatcggcaacctgctggaactcgtgggcagcgacatcgtgtccaaagaggatatcatggaagagttca
acaaatacgaccagtgcaggcccgagatcagctccatcgtgttcaacctggaaaagtgggccttcgacacataccccgagctgtctgccaga
gtggaccgggaagagaaggtggacttcaagagcatcctgaaaatcctgctgaacaacaagaacatcaacaaagagcagagcgacatcct
gcggaagatccggaacgccttcgatcacaacaattaccccgacaaaggcgtggtggaaatcaaggccctgcctgagatcgccatgagcatc
aagaaggcctttggggagtacgccatcatgaagggatcccttcaactgcctccacttgaaagactgacactggactataaggaccacgacgg
agactacaaggatcatgatattgattacaaagacgatgacgataagggcggcgggtccggaggagagggcagaggaagtctcctaacatg
cggtgacgtggaggagaatcctggcccaatgagcgagctgattaaggagaacatgcacatgaagctgtacatggagggcaccgtggacaa
ccatcacttcaagtgcacatccgagggcgaaggcaagccctacgagggcacccagaccatgagaatcaaggtggtcgagggcggccctct
cccctttcgccttcgacatcctggctactagcttcctctacggcagcaagaccttcatcaaccacacccagggcatccccgacttcttcaagcagtc
cttccctgagggcttcacatgggagagagtcaccacatacgaggacgggggcgtgctgaccgctacccaggacaccagcctccaggacgg
ctgcctcatctacaacgtcaagatcagaggggtgaacttcacatccaacggccctgtgatgcagaagaaaacactcggctgggaggccttca
ccgagaccctgtaccccgctgacggcggcctggaaggcagaaacgacatggccctgaagctcgtgggcgggagccatctgatcgcaaaca
tcaagaccacatatagatccaagaaacccgctaagaacctcaagatgcctggcgtctactatgtggactacagactggaaagaatcaagga
ggccaacaacgagacctacgtcgagcagcacgaggtggcagtggccagatactgcgacctccctagcaaactggggcacaagcttaattg
a

Black: PspCas13b

Green:3xFlag

Red: T2A tag

Blue: BFP

Coding sequence of codon optimized Spike protein-P2A-EGFP.
a t g t t c g t g t t t c t g g t g c t g c t g c c t c t g g t g a g c t c c c a g t g c g t g a a c c t g a c c a c a c -
ggacacagctgcccctgcctacaccaacagcttcacaaggggcgtgtactaccccgacaaggtgtttagatctagcgtgctgcactccacac
aggatctgtttctgcctttcttttctaacgtgacctggttccacgctatccacgtgtccggcaccaacggaacaaagaggttcgacaacccagtgct
gccctttaacgatggcgtgtacttcgcctccaccgagaagtctaacatcatcagaggctggatctttggaaccacactggacagcaagacaca
gtccctgctgatcgtgaacaacgccaccaacgtggtcatcaaggtgtgcgagttccagttttgtaacgatccattcctgggcgtgtactaccacaa
gaacaacaagtcttggatggagagcgagtttcgcgtgtactcctctgccaacaactgtacatttgagtacgtgtcccagcccttcctgatggacct
ggagggcaagcagggaaacttcaagaacctgcgggagttcgtgtttaagaacatcgatggctactttaagatctactccaagcacaccccaat
caacctggtgcgcgacctgccacagggcttctctgccctggagccactggtggatctgcccatcggaatcaacatcaccaggtttcagacactg
ctggccctgcacagaagctacctgacaccaggcgacagctcctctggatggaccgctggagctgctgcctactacgtgggctacctgcagccc
cggaccttcctgctgaagtacaacgagaacggaaccatcacagacgctgtggattgcgccctggacccctgtctgagaccaagtgtacactg
aagagctttaccgtggagaagggcatctaccagacaagcaacttccgggtgcagcctaccgagtccatcgtgcgctttcccaacatcacaaac
ctgtgcccttttggagaggtgttcaacgctacccgcttcgcctccgtgtacgcttggaaccggaagcgcatctccaactgcgtggccgactactct
gtgctgtacaacagcgccagcttcagcaccttcaagtgctacggcgtgagcccaacaaagctgaacgacctgtgctttaccaacgtgtacgctg
attccttcgtgatcaggggagacgaggtgcgccagatcgctcccggccagacaggaaagatcgctgactacaactacaagctgcctgacgat
ttcaccggctgcgtgatcgcctggaactctaacaacctggatagcaaagtgggcggaaactacaactacctgtacaggctgtttagaaagtcta
acctgaagccattcgagcgggacatctccacagagatctaccaggctggctctaccccatgcaacggagtggagggcttcaactgttacttccc
tctgcagagctacggattccagccaacaaacggcgtgggataccagccctaccgcgtggtggtgctgtcttttgagctgctgcacgctcctgcta
cagtgtgcggaccaaagaagagcaccaacctggtgaagaacaagtgcgtgaacttcaactttaacggactgaccggcacaggagtgctga
ccgagtctaacaagaagttcctgccttttcagcagttcggccgggacatcgccgataccacagacgctgtgcgcgaccctcagaccctggaga
tcctggatatcacaccatgctccttcggcggagtgtctgtgatcacaccaggaaccaacacaagcaaccaggtggccgtgctgtaccaggacg
tgaactgtaccgaggtgcccgtggctatccacgccgatcagctgacccctacatggagggtgtactctaccggcagcaacgtgttccagacaa
gagccggctgtctgatcggagctgagcacgtgaacaacagctacgagtgcgacatccctatcggcgccggaatctgtgcttcctaccagaccc
agacaaactccccaaggagagccaggtctgtggctagccagtccatcatcgcctacaccatgagcctgggcgccgagaactccgtggcttac
tccaacaactctatcgctatccctaccaacttcacaatctccgtgaccacagagatcctgccagtgagcatgaccaagacatccgtggactgca
caatgtacatctgtggagattccaccgagtgctctaacctgctgctgcagtacggctctttctgtacccagctgaacagagccctgacaggaatc
gctgtggagcaggacaagaacacacaggaggtgttcgcccaggtgaagcagatctacaagaccccacccatcaaggactttggcggattca
actttagccagatcctgcccgatcctagcaagccatccaagaggtcttttatcgaggacctgctgttcaacaaggtgaccctggctgatgccggct
tcatcaagcagtacggcgattgcctgggagacatcgctgccagagacctgatctgtgcccagaagtttaacggactgaccgtgctgcctccact
gctgacagatgagatgatcgctcagtacacatctgctctgctggccggcaccatcacaagcggatggaccttcggcgctggagctgccctgca
gatcccctttgccatgcagatggcttacagattcaacggcatcggagtgacccagaacgtgctgtacgagaaccagaagctgatcgccaacc
agtttaactccgctatcggcaagatccaggactctctgagctccacagctagcgccctgggaaagctgcaggatgtggtgaaccagaacgctc
aggccctgaacaccctggtgaagcagctgtctagcaacttcggcgccatctcctctgtgctgaacgatatcctgagcaggctggacaaggtgg
aggctgaggtgcagatcgacaggctgatcacaggaagactgcagtccctgcagacctacgtgacacagcagctgatcagggctgctgagat
cagggcttctgccaacctggctgccaccaagatgagcgagtgcgtgctgggccagtccaagagagtggacttttgtggcaagggataccacct
gatgagcttcccacagtccgcccctcacggagtggtgtttctgcacgtgacctacgtgccagctcaggagaagaacttcaccacagctcccgcc
atctgccacgatggcaaggcccactttcctcgggagggcgtgttcgtgagcaacggaacccactggtttgtgacacagcgcaacttctacgag
ccacagatcatcaccacagacaacacattcgtgtccggcaactgtgacgtggtcatcggaatcgtgaacaacaccgtgtacgatcctctgcag
ccagagctggactcttttaaggaggagctggataagtacttcaagaaccacaccagccctgacgtggatctgggcgacatctctggaatcaac
gccagcgtggtgaacatccagaaggagatcgaccggctgaacgaggtggctaagaacctgaacgagtccctgatcgatctgcaggagctg
ggcaagtacgagcagtacatcaagtggccctggtacatctggctgggcttcatcgccggactgatcgctatcgtgatggtgaccatcatgctgtg
ctgtatgacaagctgctgttcctgcctgaagggctgctgttcttgtggaagctgctgtaagtttgacgaggacgatagcgagcctgtgctgaaggg
cgtgaagctgcactacaccaagcttggatccggaagcggagctactaacttcagcctgctgaagcaggctggagacgtggaggagaaccct
ggacctatgagcaagggcgaggagctgttcaccggggtggtgcccatcctggtcgagctggacggcgacgtaaacggccacaagttcagcg
tgtccggcgagggcgagggcgatgccacctacggcaagctgaccctgaagttcatctgcaccaccggcaagctgcccgtgccctggcccac
cctcgtgaccaccctgacctacggcgtgcagtgcttcagccgctaccccgaccacatgaagcagcacgacttcttcaagtccgccatgcccga
aggctacgtccaggagcgcaccatcttcttcaaggacgacggcaactacaagacccgcgccgaggtgaagttcgagggcgacaccctggt
gaaccgcatcgagctgaagggcatcgacttcaaggaggacggcaacatcctggggcacaagctggagtacaactacaacagccacaacg
tctatatcatggccgacaagcagaagaacggcatcaaggtgaacttcaagatccgccacaacatcgaggacggcagcgtgcagctcgccg
accactaccagcagaacacccccatcggcgacggccccgtgctgctgcccgacaaccactacctgagcacccagtccgccctgagcaaag
accccaacgagaagcgcgatcacatggtcctgctggagttcgtgaccgccgccgggatcactctcggcatggacgagctgtacaagtaa

Blake: Codon-optimized Spike sequence

Blue: P2A tag

Green: EGFP

Coding sequence of Nucleocapsid phosphoprotein (NCP) tagged with 3xHA (without codon optimization).

atgtctgataatggaccccaaaatcagcgaaatgcaccccgcattacgtttggtggaccct-
cagattcaactggcagtaaccagaatggagaacgcagtggggcgcgatcaaaacaacgtcggccccaaggtttacccaataatactgcgtct
tggttcaccgctctcactcaacatggcaaggaagaccttaaattccctcgaggacaaggcgttccaattaacaccaatagcagtccagatgac
caaattggctactaccgaagagctaccagacgaattcgtggtggtgacggtaaaatgaaagatctcagtccaagatggtatttctactacctagg
aactgggccagaagctggacttccctatggtgctaacaaagacggcatcatatgggttgcaactgagggagccttgaatacaccaaaagatc
acattggcacccgcaatcctgctaacaatgctgcaatcgtgctacaacttcctcaaggaacaacattgccaaaaggcttctacgcagaaggga
gcagaggcggcagtcaagcctcttctcgttcctcatcacgtagtcgcaacagttcaagaaattcaactccaggcagcagtaggggaacttctcc
tgctagaatggctggcaatggcggtgatgctgctcttgctttgctgctgcttgacagattgaaccagcttgagagcaaaatgtctggtaaaggcca
acaacaacaaggccaaactgtcactaagaaatctgctgctgaggcttctaagaagcctcggcaaaaacgtactgccactaaagcatacaatg
taacacaagctttcggcagacgtggtccagaacaaacccaaggaaattttggggaccaggaactaatcagacaaggaactgattacaaaca
ttggccgcaaattgcacaatttgcccccagcgcttcagcgttcttcggaatgtcgcgcattggcatggaagtcacaccttcgggaacgtggttgac
ctacacaggtgccatcaaattggatgacaaagatccaaatttcaaagatcaagtcattttgctgaataagcatattgacgcatacaaaacattcc
caccaacagagcctaaaaaggacaaaaagaagaaggctgatgaaactcaagccttaccgcagagacagaagaaacagcaaactgtga
ctcttcttcctgctgcagatttggatgatttctccaaacaattgcaacaatccatgagcagtgctgactcaactcaggccggtagttcctacccatac
gatgttccagattacgcttatccctacgacgtgcctgattatgcatacccatatgatgtccccgactatgcctaa

Black: Nucleocapsid protein
Green: 3xHA tag

Partial coding sequence of Spike D614G tagged with 3xHA (without codon optimization).

atgtttgtaattagaggtgatgaagtcagacaaatcgctccagggcaaactg-
gaaagattgctgattataattataaattaccagatgattttacaggctgcgttatagcttggaattctaacaatcttgattctaaggttggtggtaattat
aattacctgtatagattgtttaggaagtctaatctcaaaccttttgagagagatatttcaactgaaatctatcaggccggtagcacaccttgtaatggt
gttgaaggttttaattgttactttcctttacaatcatatggtttccaacccactaatggtgttggttaccaaccatacagagtagtagtactttcttttgaact
tctacatgcaccagcaactgtttgtggacctaaaaagtctactaatttggttaaaaacaaatgtgtcaatttcaacttcaatggtttaacaggcacag
gtgttcttactgagtctaacaaaaagtttctgcctttccaacaatttggcagagacattgctgacactactgatgctgtccgtgatccacagacacttg
agattcttgacattacaccatgttcttttggtggtgtcagtgttataacaccaggaacaaatacttctaaccaggttgctgttctttatcagggtgttaact
gcacagaagtccctgttgctattcatgcagatcaacttactcctacttggcgtgtttattctacaggttctaatgtttttcaaacacgtgcaggctgttta
ataggggctgaacatgtcaacaactcatatgagtgtgacatacccattggtgcaggtatatgcgctagttatcagactcagactaattctcctcgg
cgggcacgtagtgtagctagtcaatccatcattgcctacactatgtcacttggtgcagaaaattcagttgcttactctaataactctattgccataccc
acaaattttactattagtgttaccacagaaattctaccagtgtctatgaccaagacatcagtagattgtacaatgtacatttgtggtgattcaactgaa
tgcagcaatcttttgttgcaatatggcagtttttgtacacaattaaaccgtgctttaactggaatagctgttgaacaagacaaaaacacccaagaa
gtttttgcacaagtcaaacaaatttacaaaacaccaccaattaaagattttggtggttttaatttttcacaaatattggtagttcctacccatacgatgtt
ccagattacgcttatccctacgacgtgcctgattatgcatacccatatgatgtccccgactatgcctaa

Black: part of the Spike sequence
Green: 3xHA tag
