# Supplementary material for: Reprogrammed CRISPR-Cas13b suppresses SARS-CoV-2 replication and circumvents its mutational escape through mismatch tolerance
Source: Nat Commun. 2021 Jul 13;12:4270. doi: 10.1038/s41467-021-24577-9 (PMC8277810; doi:10.1038/s41467-021-24577-9)
Supplement: Supplementary file 6 — Supplementary Data file 5 [file 41467_2021_24577_MOESM6_ESM.docx]

| Number of mismatches (MSM) with targets in the human transcriptome | crRNAs with potential off-targets |
| --- | --- |
| 1 | 0 |
| 2 | 0 |
| 3 | 6 |
| 4 | 21 |
| 5 | 61 |
| 6 | 344 |
| **Total crRNA in the final list**  **(crRNAs with 6-nt or less MSM with human transcriptome are excluded from the list)** | **495** |

**Supplementary Table 5.**
