# Supplementary material for: Reprogrammed CRISPR-Cas13b suppresses SARS-CoV-2 replication and circumvents its mutational escape through mismatch tolerance
Source: Nat Commun. 2021 Jul 13;12:4270. doi: 10.1038/s41467-021-24577-9 (PMC8277810; doi:10.1038/s41467-021-24577-9)
Supplement: Supplementary file 9 — Supplementary Data file 8 [file 41467_2021_24577_MOESM9_ESM.docx]

**Supplementary Table 8**

| Guide RNA name | crRNA Target/Description | Sequence | Figure |
| --- | --- | --- | --- |
| NT | Non targeting crRNA | TAGATTGCTGTTCTACCAAGTAATCCATCA | Figure1-5 |
| Spike crRNA1 | crRNA targeting Spike (codon optimised) | GTTAGAAAAGAAAGGCAGAAACAGATCCTG | Figure1 |
| Spike crRNA2 | crRNA targeting Spike (codon optimised) | GGAGTAGATCTTAAAGTAGCCATCGATGTT | Figure1 |
| Spike crRNA3 | crRNA targeting Spike (codon optimised) | GTCCTCGATAAAAGACCTCTTGGATGGCTT | Figure1 |
| Spike crRNA4 | crRNA targeting Spike (codon optimised) | GTTCTTGAAGTACTTATCCAGCTCCTCCTT | Figure1 |
| Spike crRNA -TIL1 | Tiled crRNA targeting Spike | GTCGCGCACCAGGTTGATTGGGGTGTGCTT | Figure1 |
| Spike crRNA-TIL2 | Tiled crRNA targeting Spike | TCGCGCACCAGGTTGATTGGGGTGTGCTTG | Figure1 |
| Spike crRNA-TIL3 | Tiled crRNA targeting Spike | CGCGCACCAGGTTGATTGGGGTGTGCTTGG | Figure1 |
| Spike crRNA-TIL4 | Tiled crRNA targeting Spike | GCGCACCAGGTTGATTGGGGTGTGCTTGGA | Figure1 |
| Spike crRNA-TILF5 | Tiled crRNA targeting Spike | CGCACCAGGTTGATTGGGGTGTGCTTGGAG | Figure1 |
| Spike crRNA-TIL6 | Tiled crRNA targeting Spike | GCACCAGGTTGATTGGGGTGTGCTTGGAGT | Figure1 |
| Spike crRNA-TIL7 | Tiled crRNA targeting Spike | CACCAGGTTGATTGGGGTGTGCTTGGAGTA | Figure1 |
| Spike crRNA-TIL8 | Tiled crRNA targeting Spike | ACCAGGTTGATTGGGGTGTGCTTGGAGTAG | Figure1 |
| Spike crRNA-TILF9 | Tiled crRNA targeting Spike | CCAGGTTGATTGGGGTGTGCTTGGAGTAGA | Figure1 |
| Spike crRNA-TIL10 | Tiled crRNA targeting Spike | CAGGTTGATTGGGGTGTGCTTGGAGTAGAT | Figure1 |
| Spike crRNA-TIL11 | Tiled crRNA targeting Spike | AGGTTGATTGGGGTGTGCTTGGAGTAGATC | Figure1 |
| Spike crRNA-TIL12 | Tiled crRNA targeting Spike | GGTTGATTGGGGTGTGCTTGGAGTAGATCT | Figure1 |
| Spike crRNA-TIL13 | Tiled crRNA targeting Spike | GTTGATTGGGGTGTGCTTGGAGTAGATCTT | Figure1 |
| Spike crRNA-TIL14 | Tiled crRNA targeting Spike | TTGATTGGGGTGTGCTTGGAGTAGATCTTA | Figure1 |
| Spike crRNA-TIL15 | Tiled crRNA targeting Spike | TGATTGGGGTGTGCTTGGAGTAGATCTTAA | Figure1 |
| Spike crRNA-TIL16 | Tiled crRNA targeting Spike | GATTGGGGTGTGCTTGGAGTAGATCTTAAA | Figure1 |
| Spike crRNA-TIL17 | Tiled crRNA targeting Spike | ATTGGGGTGTGCTTGGAGTAGATCTTAAAG | Figure1 |
| Spike crRNA-TIL18 | Tiled crRNA targeting Spike | TTGGGGTGTGCTTGGAGTAGATCTTAAAGT | Figure1 |
| Spike crRNA-TIL19 | Tiled crRNA targeting Spike | TGGGGTGTGCTTGGAGTAGATCTTAAAGTA | Figure1 |
| Spike crRNA-TIL20 | Tiled crRNA targeting Spike | GGGGTGTGCTTGGAGTAGATCTTAAAGTAG | Figure1 |
| Spike crRNA-TIL21 | Tiled crRNA targeting Spike | GGGTGTGCTTGGAGTAGATCTTAAAGTAGC | Figure1 |
| Spike crRNA-TIL22 | Tiled crRNA targeting Spike | GGTGTGCTTGGAGTAGATCTTAAAGTAGCC | Figure1 |
| Spike crRNA-TIL23 | Tiled crRNA targeting Spike | GTGTGCTTGGAGTAGATCTTAAAGTAGCCA | Figure1 |
| Spike crRNA-TIL24 | Tiled crRNA targeting Spike | TGTGCTTGGAGTAGATCTTAAAGTAGCCAT | Figure1 |
| Spike crRNA-TIL25 | Tiled crRNA targeting Spike | GTGCTTGGAGTAGATCTTAAAGTAGCCATC | Figure1 |
| Spike crRNA-TIL26 | Tiled crRNA targeting Spike | TGCTTGGAGTAGATCTTAAAGTAGCCATCG | Figure1 |
| Spike crRNA-TIL27 | Tiled crRNA targeting Spike | GCTTGGAGTAGATCTTAAAGTAGCCATCGA | Figure1 |
| Spike crRNA-TIL28 | Tiled crRNA targeting Spike | CTTGGAGTAGATCTTAAAGTAGCCATCGAT | Figure1 |
| Spike crRNA-TIL29 | Tiled crRNA targeting Spike | TTGGAGTAGATCTTAAAGTAGCCATCGATG | Figure1 |
| Spike crRNA-TIL30 | Tiled crRNA targeting Spike | TGGAGTAGATCTTAAAGTAGCCATCGATGT | Figure1 |
| Spike crRNA-TIL31 | Tiled crRNA targeting Spike | GGAGTAGATCTTAAAGTAGCCATCGATGTT | Figure1 |
| Spike crRNA-TIL32 | Tiled crRNA targeting Spike | GAGTAGATCTTAAAGTAGCCATCGATGTTC | Figure1 |
| Spike crRNA-TIL33 | Tiled crRNA targeting Spike | AGTAGATCTTAAAGTAGCCATCGATGTTCT | Figure1 |
| Spike crRNA-TIL34 | Tiled crRNA targeting Spike | GTAGATCTTAAAGTAGCCATCGATGTTCTT | Figure1 |
| Spike crRNA-TIL35 | Tiled crRNA targeting Spike | TAGATCTTAAAGTAGCCATCGATGTTCTTA | Figure1 |
| Spike crRNA-TIL36 | Tiled crRNA targeting Spike | AGATCTTAAAGTAGCCATCGATGTTCTTAA | Figure1 |
| Spike crRNA-TIL37 | Tiled crRNA targeting Spike | GATCTTAAAGTAGCCATCGATGTTCTTAAA | Figure1 |
| Spike crRNA-TIL38 | Tiled crRNA targeting Spike | ATCTTAAAGTAGCCATCGATGTTCTTAAAC | Figure1 |
| Spike crRNA-TIL39 | Tiled crRNA targeting Spike | TCTTAAAGTAGCCATCGATGTTCTTAAACA | Figure1 |
| Spike crRNA-TIL40 | Tiled crRNA targeting Spike | CTTAAAGTAGCCATCGATGTTCTTAAACAC | Figure1 |
| Spike crRNA-TIL41 | Tiled crRNA targeting Spike | TTAAAGTAGCCATCGATGTTCTTAAACACG | Figure1 |
| Spike crRNA-TIL42 | Tiled crRNA targeting Spike | TAAAGTAGCCATCGATGTTCTTAAACACGA | Figure1 |
| Spike crRNA-TIL43 | Tiled crRNA targeting Spike | AAAGTAGCCATCGATGTTCTTAAACACGAA | Figure1 |
| Spike crRNA-TIL44 | Tiled crRNA targeting Spike | AAGTAGCCATCGATGTTCTTAAACACGAAC | Figure1 |
| Spike crRNA-TIL45 | Tiled crRNA targeting Spike | AGTAGCCATCGATGTTCTTAAACACGAACT | Figure1 |
| Spike crRNA-TIL46 | Tiled crRNA targeting Spike | GTAGCCATCGATGTTCTTAAACACGAACTC | Figure1 |
| Spike crRNA-TIL47 | Tiled crRNA targeting Spike | TAGCCATCGATGTTCTTAAACACGAACTCC | Figure1 |
| Spike crRNA-TIL48 | Tiled crRNA targeting Spike | AGCCATCGATGTTCTTAAACACGAACTCCC | Figure1 |
| Spike crRNA-TIL49 | Tiled crRNA targeting Spike | GCCATCGATGTTCTTAAACACGAACTCCCG | Figure1 |
| Spike crRNA-TIL50 | Tiled crRNA targeting Spike | CCATCGATGTTCTTAAACACGAACTCCCGC | Figure1 |
| Spike crRNA-TIL51 | Tiled crRNA targeting Spike | CATCGATGTTCTTAAACACGAACTCCCGCA | Figure1 |
| Spike crRNA-TIL52 | Tiled crRNA targeting Spike | ATCGATGTTCTTAAACACGAACTCCCGCAG | Figure1 |
| Spike crRNA-TIL53 | Tiled crRNA targeting Spike | TCGATGTTCTTAAACACGAACTCCCGCAGG | Figure1 |
| Spike crRNA-TIL54 | Tiled crRNA targeting Spike | CGATGTTCTTAAACACGAACTCCCGCAGGT | Figure1 |
| Spike crRNA-TIL55 | Tiled crRNA targeting Spike | GATGTTCTTAAACACGAACTCCCGCAGGTT | Figure1 |
| Spike crRNA-TIL56 | Tiled crRNA targeting Spike | ATGTTCTTAAACACGAACTCCCGCAGGTTC | Figure1 |
| Spike crRNA-TIL57 | Tiled crRNA targeting Spike | TGTTCTTAAACACGAACTCCCGCAGGTTCT | Figure1 |
| Spike crRNA-TIL58 | Tiled crRNA targeting Spike | GTTCTTAAACACGAACTCCCGCAGGTTCTT | Figure1 |
| Spike crRNA-TIL59 | Tiled crRNA targeting Spike | TTCTTAAACACGAACTCCCGCAGGTTCTTG | Figure1 |
| Spike crRNA-TIL60 | Tiled crRNA targeting Spike | TCTTAAACACGAACTCCCGCAGGTTCTTGA | Figure1 |
| Spike crRNA-TIL61 | Tiled crRNA targeting Spike | CTTAAACACGAACTCCCGCAGGTTCTTGAA | Figure1 |
| Spike crRNA2_1-3-nt MSM | Mutagenesis of Spike crRNA2 at positions 1-3 | CCTGTAGATCTTAAAGTAGCCATCGATGTT | Figure1 |
| Spike crRNA2_1-6-nt MSM | Mutagenesis of Spike crRNA2 at positions 1-6 | CCTCATGATCTTAAAGTAGCCATCGATGTT | Figure1 |
| Spike crRNA2_1-9-nt MSM | Mutagenesis of Spike crRNA2 at positions 1-9 | CCTCATCTACTTAAAGTAGCCATCGATGTT | Figure1 |
| Spike crRNA2_28-30-nt MSM | Mutagenesis of Spike crRNA2 at positions 28-30 | GGAGTAGATCTTAAAGTAGCCATCGATCAA | Figure1 |
| Spike crRNA2_25-30-nt MSM | Mutagenesis of Spike crRNA2 at positions 25-30 | GGAGTAGATCTTAAAGTAGCCATCCTACAA | Figure1 |
| Spike crRNA2_22-30-nt MSM | Mutagenesis of Spike crRNA2 at positions 22-30 | GGAGTAGATCTTAAAGTAGCCTAGCTACAA | Figure1 |
| Spike crRNA2_14-16-nt MSM | Mutagenesis of Spike crRNA2 at positions 14-16 | GGAGTAGATCTTATTCTAGCCATCGATGTT | Figure1 |
| Spike crRNA2_13-18-nt MSM | Mutagenesis of Spike crRNA2 at positions 13-18 | GGAGTAGATCTTTTTCATGCCATCGATGTT | Figure1 |
| Spike crRNA2_11-19-nt MSM | Mutagenesis of Spike crRNA2 at positions 11-19 | GGAGTAGATCAATTTCATCCCATCGATGTT | Figure1 |
| Spike crRNA1_NCO | crRNA targeting Spike (not codon optimised) | GTCAGGGTAATAAACACCACGTGTGAAAGA | Figure4 |
| Spike crRNA2 NCO | crRNA targeting Spike (not codon optimised) | GGCAAATCTACCAATGGTTCTAAAGCCGAA | Figure4 |
| Spike crRNA3 NCO | crRNA targeting Spike (not codon optimised) | GAAGCATTAATGCCAGAGATGTCACCTAAA | Figure4 |
| Spike crRNA4 NCO | crRNA targeting Spike (not codon optimised) | GCACTGGCTCAGAGTCGTCTTCATCAAATT | Figure4 |
| NCP crRNA1 | crRNA targeting Nucleocapsid protein (NCP) | GTCTTCCTTGCCATGTTGAGTGAGAGCGGT | Figure2,4,5 |
| NCP crRNA2 | crRNA targeting Nucleocapsid protein (NCP) | GACTGAGATCTTTCATTTTACCGTCACCAC | Figure2&4 |
| NCP gRAN3 | crRNA targeting Nucleocapsid protein (NCP) | GAATTTCTTGAACTGTTGCGACTACGTGAT | Figure2&4 |
| NCP crRNA4 | crRNA targeting Nucleocapsid protein (NCP) | GTTCAATCTGTCAAGCAGCAGCAAAGCAAG | Figure2&4 |
| NCP crRNA1_1-3-nt MSM | Mutagenesis of NCP crRNA1 at positions 1-3 | CAGTTCCTTGCCATGTTGAGTGAGAGCGGT | Figure2 |
| NCP crRNA1_1-6-nt MSM | Mutagenesis of NCP crRNA1 at positions 1-6 | CAGAAGCTTGCCATGTTGAGTGAGAGCGGT | Figure2 |
| NCP crRNA1_1-9-nt MSM | Mutagenesis of NCP crRNA1 at positions 1-9 | CAGAAGGAAGCCATGTTGAGTGAGAGCGGT | Figure2 |
| NCP crRNA1_1-12-nt MSM | Mutagenesis of NCP crRNA1 at positions 1-12 | CAGAAGGAACGGATGTTGAGTGAGAGCGGT | Figure2 |
| NCP crRNA1_1-15-nt MSM | Mutagenesis of NCP crRNA1 at positions 1-15 | CAGAAGGAACGGTACTTGAGTGAGAGCGGT | Figure2 |
| NCP crRNA1_1-18-nt MSM | Mutagenesis of NCP crRNA1 at positions 1-18 | CAGAAGGAACGGTACAACAGTGAGAGCGGT | Figure2 |
| NCP crRNA1_1-21-nt MSM | Mutagenesis of NCP crRNA1 at positions 1-21 | CAGAAGGAACGGTACAACTCAGAGAGCGGT | Figure2 |
| NCP crRNA1_1-24-nt MSM | Mutagenesis of NCP crRNA1 at positions 1-24 | CAGAAGGAACGGTACAACTCACTCAGCGGT | Figure2 |
| NCP crRNA1_1-27-nt MSM | Mutagenesis of NCP crRNA1 at positions 1-27 | CAGAAGGAACGGTACAACTCACTCTCGGGT | Figure2 |
| NCP crRNA1_1-30-nt MSM | Mutagenesis of NCP crRNA1 at positions 1-30 | CAGAAGGAACGGTACAACTCACTCTCGCCA | Figure2 |
| NCP crRNA1_14-16-nt MSM | Mutagenesis of NCP crRNA1 at positions 14-16 | GTCTTCCTTGCCAACATGAGTGAGAGCGGT | Figure2 |
| NCP crRNA1_13-18-nt MSM | Mutagenesis of NCP crRNA1 at positions 13-18 | GTCTTCCTTGCCTACAACAGTGAGAGCGGT | Figure2 |
| NCP crRNA1_11-19-nt MSM | Mutagenesis of NCP crRNA1 at positions 11-19 | GTCTTCCTTGGGTACAACTGTGAGAGCGGT | Figure2 |
| NCP crRNA1_10-21-nt MSM | Mutagenesis of NCP crRNA1 at positions 10-21 | GTCTTCCTTCGGTACAACTCAGAGAGCGGT | Figure2 |
| NCP crRNA1_8-22-nt MSM | Mutagenesis of NCP crRNA1 at positions 8-22 | GTCTTCCAACGGTACAACTCACAGAGCGGT | Figure2 |
| NCP crRNA1_7-24-nt MSM | Mutagenesis of NCP crRNA1 at positions 7-24 | GTCTTCGAACGGTACAACTCACTCAGCGGT | Figure2 |
| NCP crRNA1_5-25-nt MSM | Mutagenesis of NCP crRNA1 at positions 5-25 | GTCTAGGAACGGTACAACTCACTCTGCGGT | Figure2 |
| NCP crRNA1_4-27-nt MSM | Mutagenesis of NCP crRNA1 at positions 4-27 | GTCAAGGAACGGTACAACTCACTCTCGGGT | Figure2 |
| NCP crRNA1_2-28-nt MSM | Mutagenesis of NCP crRNA1 at positions 2-28 | GAGAAGGAACGGTACAACTCACTCTCGCGT | Figure2 |
| NCP crRNA1_2-30-nt MSM | Mutagenesis of NCP crRNA1 at positions 2-30 | GAGAAGGAACGGTACAACTCACTCTCGCCA | Figure2 |
| NCP crRNA1_28-30-nt MSM | Mutagenesis of NCP crRNA1 at positions 28-30 | GTCTTCCTTGCCATGTTGAGTGAGAGCCCA | Figure2 |
| NCP crRNA1_25-30-nt MSM | Mutagenesis of NCP crRNA1 at positions 25-30 | GTCTTCCTTGCCATGTTGAGTGAGTCGCCA | Figure2 |
| NCP crRNA1_22-30-nt MSM | Mutagenesis of NCP crRNA1 at positions 22-30 | GTCTTCCTTGCCATGTTGAGTCTCTCGCCA | Figure2 |
| NCP crRNA1_19-30-nt MSM | Mutagenesis of NCP crRNA1 at positions 19-30 | GTCTTCCTTGCCATGTTGTCACTCTCGCCA | Figure2 |
| NCP crRNA1_16-30-nt MSM F | Mutagenesis of NCP crRNA1 at positions 16-30 | GTCTTCCTTGCCATGAACTCACTCTCGCCA | Figure2 |
| NCP crRNA1_13-30-nt MSM | Mutagenesis of NCP crRNA1 at positions 13-30 | GTCTTCCTTGCCTACAACTCACTCTCGCCA | Figure2 |
| NCP crRNA1_10-30-nt MSM | Mutagenesis of NCP crRNA1 at positions 10-30 | GTCTTCCTTCGGTACAACTCACTCTCGCCA | Figure2 |
| NCP crRNA1_7-30-nt MSM | Mutagenesis of NCP crRNA1 at positions 7-30 | GTCTTCGAACGGTACAACTCACTCTCGCCA | Figure2 |
| NCP crRNA1_4-30-nt MSM | Mutagenesis of NCP crRNA1 at positions 4-30 | GTCAAGGAACGGTACAACTCACTCTCGCCA | Figure2 |
| NSP7 crRNA1 | Non-structural protein 7 | GATGATGATTCTACTCTGAGTTGTTGCAAA | Figure4 |
| NSP7 crRNA2 | Non-structural protein 7 | GGAAAGCAAAACAGAAAGTAGTGAAACCAT | Figure4 |
| NSP7 crRNA3 | Non-structural protein 7 | GCTTGTTTATGTCTACAGCACCCTGCATGG | Figure4 |
| NSP7 crRNA4 | Non-structural protein 7 | GTTGCCCTGTTGTCCAGCATTTCTTCACAA | Figure4 |
| NSP8 crRNA1 | Non-structural protein 8 | GAACAACTTCAGAATCACCATTAGCAACAG | Figure4 |
| NSP8 crRNA2 | Non-structural protein 8 | GCATGGCTGCATCACGGTCAAATTCAGATT | Figure4 |
| NSP8 crRNA3 | Non-structural protein 8 | GCTTGATCAGCCATCTTTTCCAACTTACGT | Figure4 |
| NSP8 crRNA4 | Non-structural protein 8 | GCATCATTATCCAACTTTCTAAGCATAGTG | Figure4 |
|  |  |  |  |
| Spike crRNA2_1st_MSM | Mutagenesis of Spike crRNA2 at position 1 | CGAGTAGATCTTAAAGTAGCCATCGATGTT | Figure3 |
| Spike crRNA 2_5th_MSM | Mutagenesis of Spike crRNA2 at position 5 | GGAGAAGATCTTAAAGTAGCCATCGATGTT | Figure3 |
| Spike crRNA 2_10th_MSM | Mutagenesis of Spike crRNA2 at position 10 | GGAGTAGATGTTAAAGTAGCCATCGATGTT | Figure3 |
| Spike crRNA 2_15th_MSM | Mutagenesis of Spike crRNA2 at position 15 | GGAGTAGATCTTAATGTAGCCATCGATGTT | Figure3 |
| Spike crRNA 2_20th_MSM | Mutagenesis of Spike crRNA2 at position 20 | GGAGTAGATCTTAAAGTAGGCATCGATGTT | Figure3 |
| Spike crRNA 2_25th_MSM | Mutagenesis of Spike crRNA2 at position 25 | GGAGTAGATCTTAAAGTAGCCATCCATGTT | Figure3 |
| Spike crRNA 2_30th_MSM | Mutagenesis of Spike crRNA2 at position 30 | GGAGTAGATCTTAAAGTAGCCATCGATGTA | Figure3 |
| NCP crRNA1_1st_MSM | Mutagenesis of NCP crRNA1 at position 1 | CTCTTCCTTGCCATGTTGAGTGAGAGCGGT | Figure3 |
| NCP crRNA1_5th_MSM | Mutagenesis of NCP crRNA1 at position 5 | GTCTACCTTGCCATGTTGAGTGAGAGCGGT | Figure3 |
| NCP crRNA1_10th_MSM | Mutagenesis of NCP crRNA1 at position 10 | GTCTTCCTTCCCATGTTGAGTGAGAGCGGT | Figure3 |
| NCP crRNA1_15th_MSM | Mutagenesis of NCP crRNA1 at position 15 | GTCTTCCTTGCCATCTTGAGTGAGAGCGGT | Figure3 |
| NCP crRNA1_20th_MSM | Mutagenesis of NCP crRNA1 at position 20 | GTCTTCCTTGCCATGTTGACTGAGAGCGGT | Figure3 |
| NCP crRNA1_25th_MSM | Mutagenesis of NCP crRNA1 at position 25 | GTCTTCCTTGCCATGTTGAGTGAGTGCGGT | Figure3 |
| NCP crRNA1_30th_MSM | Mutagenesis of NCP crRNA1 at position30 | GTCTTCCTTGCCATGTTGAGTGAGAGCGGA | Figure3 |
| D614G targeting crRNA with MSM at G–U position 5 | crRNA targeting Spike D614 genomic region. Full match with the ancestral SARS-CoV-2 and 1 MSM with the D614G mutant at spacer position 5 (not codon-optimized) | AACATCCTGATAAAGAACAGCAACCTGGTT | Figure5&6 |
| D614G targeting crRNA with MSM at G–U position 10 | crRNA targeting Spike D614 genomic region. Full match with the ancestral SARS-CoV-2 and 1 MSM with the D614G mutant at spacer position 10 (not codon-optimized) | CAGTTAACATCCTGATAAAGAACAGCAACC | Figure5&6 |
| D614G targeting crRNA with MSM at G–U position 15 | crRNA targeting Spike D614 genomic region. Full match with the ancestral SARS-CoV-2 and 1 MSM with the D614G mutant at spacer position 15 (not codon-optimized) | CTGTGCAGTTAACATCCTGATAAAGAACAG | Figure5&6 |
| D614G targeting crRNA with MSM at G–U position 20 | crRNA targeting Spike D614 genomic region. Full match with the ancestral SARS-CoV-2 and 1 MSM with the D614G mutant at spacer position 20 (not codon-optimized) | GACTTCTGTGCAGTTAACATCCTGATAAAG | Figure5&6 |
| D614G targeting crRNA with MSM at G–U position 25 | crRNA targeting Spike D614 genomic region. Full match with the ancestral SARS-CoV-2 and 1 MSM with the D614G mutant at spacer position 25 (not codon-optimized) | ACAGGGACTTCTGTGCAGTTAACATCCTGA | Figure5&6 |
| D614G targeting crRNA with G–U MSM at position 30 | crRNA targeting Spike D614 genomic region. Full match with the ancestral SARS-CoV-2 and 1 MSM with the D614G mutant at spacer position 30 (not codon-optimized) | TAGCAACAGGGACTTCTGTGCAGTTAACAT | Figure5&6 |
| D614G targeting crRNA with G–C full match at position 5 | crRNA targeting Spike D614G genomic region with saper-target full match (not codon-optimized) | AACACCCTGATAAAGAACAGCAACCTGGTT | Figure5 |
| D614G targeting crRNA with G–C full match at position 10 | crRNA targeting Spike D614G genomic region with saper-target full match (not codon-optimized) | CAGTTAACACCCTGATAAAGAACAGCAACC | Figure5 |
| D614G targeting crRNA with G–C full match at position 15 | crRNA targeting Spike D614G genomic region with saper-target full match (not codon-optimized) | CTGTGCAGTTAACACCCTGATAAAGAACAG | Figure5 |
| D614G targeting crRNA with G–C full match at position 20 | crRNA targeting Spike D614G genomic region with saper-target full match (not codon-optimized) | GACTTCTGTGCAGTTAACACCCTGATAAAG | Figure5 |
| D614G targeting crRNA with G–C full match at position 25 | crRNA targeting Spike D614G genomic region with saper-target full match (not codon-optimized) | ACAGGGACTTCTGTGCAGTTAACACCCTGA | Figure5 |
| D614G targeting crRNA with G–C full match at position 30 | crRNA targeting Spike D614G genomic region with saper-target full match (not codon-optimized) | TAGCAACAGGGACTTCTGTGCAGTTAACAC | Figure5 |
| D614G targeting crRNA with G–G MSM at position 5 | crRNA targeting Spike D614 genomic region. Full match with the ancestral SARS-CoV-2 and 1 MSM with the D614G mutant at spacer position 5 (not codon-optimized) | AACAGCCTGATAAAGAACAGCAACCTGGTT | Figure5 |
| D614G targeting crRNA with G–G MSM at position 10 | crRNA targeting Spike D614 genomic region. Full match with the ancestral SARS-CoV-2 and 1 MSM with the D614G mutant at spacer position 10 (not codon-optimized) | CAGTTAACAGCCTGATAAAGAACAGCAACC | Figure5 |
| D614G targeting crRNA with G–G MSM at position 15 | crRNA targeting Spike D614 genomic region. Full match with the ancestral SARS-CoV-2 and 1 MSM with the D614G mutant at spacer position 15 (not codon-optimized) | CTGTGCAGTTAACAGCCTGATAAAGAACAG | Figure5 |
| D614G targeting crRNA with G–G MSM at position 20 | crRNA targeting Spike D614 genomic region. Full match with the ancestral SARS-CoV-2 and 1 MSM with the D614G mutant at spacer position 20 (not codon-optimized) | GACTTCTGTGCAGTTAACAGCCTGATAAAG | Figure5 |
| D614G targeting crRNA with G–G MSM at position 25 | crRNA targeting Spike D614 genomic region. Full match with the ancestral SARS-CoV-2 and 1 MSM with the D614G mutant at spacer position 25 (not codon-optimized) | ACAGGGACTTCTGTGCAGTTAACAGCCTGA | Figure5 |
| D614G targeting crRNA with G–G MSM at position 30 | crRNA targeting Spike D614 genomic region. Full match with the ancestral SARS-CoV-2 and 1 MSM with the D614G mutant at spacer position 30 (not codon-optimized) | TAGCAACAGGGACTTCTGTGCAGTTAACAG | Figure5 |
| NCP crRNA-5T-1 | crRNA targeting NCP transcript with 5 succissive T bases in the spacer | TTTTTAGGCTCTGTTGGTGGGAATGTTTTG | Suppl. Figure 6 |
| NCP crRNA-5T-2 | crRNA targeting NCP transcript with 5 succissive T bases in the spacer | TTTTTGTCCTTTTTAGGCTCTGTTGGTGGG | Suppl. Figure 6 |
| NCP crRNA-5T-3 | crRNA targeting NCP transcript with 5 succissive T bases in the spacer | TTTTTGCCGAGGCTTCTTAGAAGCCTCAGC | Suppl. Figure 6 |
| NCP crRNA-5T-4 | crRNA targeting NCP transcript with 5 succissive T bases in the spacer | AGTGGCAGTACGTTTTTGCCGAGGCTTCTT | Suppl. Figure 6 |
| NCP crRNA-4T-1 | crRNA targeting NCP transcript with 4 succissive T bases in the spacer | TTTTGATCGCGCCCCACTGCGTTCTCCATT | Suppl. Figure 6 |
| NCP crRNA-4T-2 | crRNA targeting NCP transcript with 4 succissive T bases in the spacer | GGCCGACGTTGTTTTGATCGCGCCCCACTG | Suppl. Figure 6 |
| NCP crRNA-4T-3 | crRNA targeting NCP transcript with 4 succissive T bases in the spacer | TTTTACCGTCACCACCACGAATTCGTCTGG | Suppl. Figure 6 |
| NCP crRNA-4T-4 | crRNA targeting NCP transcript with 4 succissive T bases in the spacer | GAGATCTTTCATTTTACCGTCACCACCACG | Suppl. Figure 6 |
| NCP crRNA-4T-5 | crRNA targeting NCP transcript with 4 succissive T bases in the spacer | TTTTGGTGTATTCAAGGCTCCCTCAGTTGC | Suppl. Figure 6 |
| NCP crRNA-4T-6 | crRNA targeting NCP transcript with 4 succissive T bases in the spacer | GCCAATGTGATCTTTTGGTGTATTCAAGGC | Suppl. Figure 6 |
| NCP crRNA-4T-7 | crRNA targeting NCP transcript with 4 succissive T bases in the spacer | TTTTGGCAATGTTGTTCCTTGAGGAAGTTG | Suppl. Figure 6 |
| NCP crRNA-4T-8 | crRNA targeting NCP transcript with 4 succissive T bases in the spacer | GCGTAGAAGCCTTTTGGCAATGTTGTTCCT | Suppl. Figure 6 |
| NCP crRNA-4C-1 | crRNA targeting NCP transcript with 4 succissive C bases in the spacer | CCCCAAAATTTCCTTGGGTTTGTTCTGGAC | Suppl. Figure 6 |
| NCP crRNA-4C-2 | crRNA targeting NCP transcript with 4 succissive C bases in the spacer | GTTCCTGGTCCCCAAAATTTCCTTGGGTTT | Suppl. Figure 6 |
| NCP crRNA-4C-3 | crRNA targeting NCP transcript with 4 succissive C bases in the spacer | GTTCCTTGTCTGATTAGTTCCTGGTCCCCA | Suppl. Figure 6 |
| NCP crRNA-4C-4 | crRNA targeting NCP transcript with 4 succissive C bases in the spacer | CCCCTACTGCTGCCTGGAGTTGAATTTCTT | Suppl. Figure 6 |
| NCP crRNA-4C-5 | crRNA targeting NCP transcript with 4 succissive C bases in the spacer | GCAGGAGAAGTTCCCCTACTGCTGCCTGGA | Suppl. Figure 6 |
| NCP crRNA-4C-6 | crRNA targeting NCP transcript with 4 succissive C bases in the spacer | GCCAGCCATTCTAGCAGGAGAAGTTCCCCT | Suppl. Figure 6 |
| NCP crRNA-4C-7 | crRNA targeting NCP transcript with 4 succissive C bases in the spacer | CCCCACTGCGTTCTCCATTCTGGTTACTGC | Suppl. Figure 6 |
| NCP crRNA-4C-8 | crRNA targeting NCP transcript with 4 succissive C bases in the spacer | GTTTTGATCGCGCCCCACTGCGTTCTCCAT | Suppl. Figure 6 |
| NCP crRNA-4C-9 | crRNA targeting NCP transcript with 4 succissive C bases in the spacer | TGGGGCCGACGTTGTTTTGATCGCGCCCC | Suppl. Figure 6 |
