# Supplementary material for: Reprogrammed CRISPR-Cas13b suppresses SARS-CoV-2 replication and circumvents its mutational escape through mismatch tolerance
Source: Nat Commun. 2021 Jul 13;12:4270. doi: 10.1038/s41467-021-24577-9 (PMC8277810; doi:10.1038/s41467-021-24577-9)
Supplement: Supplementary file 10 — Supplementary Data file 9 [file 41467_2021_24577_MOESM10_ESM.docx]

| **Component (per well)** | **96-well** | **24-well** | **6-well** |
| --- | --- | --- | --- |
| # of seeded HEK 293T cells | 30,000 | 150,000 | 750,000 |
| Plasmids DNA amount | 100ng | 500ng | 2500ng |
| P3000 reagent | 0.2μL | 1μL | 5μL |
| Lipofectamine 3000 reagent | 0.3μL | 1.5μL | 7.5μL |
| Opti-MEM | Up to 10 μL | Up to 50 μL | Up to 250 μL |

**Supplementary Data file 9**. Transfection conditions of HEK 293T, VERO, and Calu-3 cell lines.

| **Component (per well)** | **96-well** | **24-well** | **6-well** |
| --- | --- | --- | --- |
| # of seeded VERO/Calu-3 cells | 20,000 | 100,000 | 500,000 |
| Plasmids DNA amount | 200ng | 1000ng | 5000ng |
| P3000 reagent | 0.4μL | 2μL | 10μL |
| Lipofectamine 3000 reagent | 0.6μL | 3μL | 15μL |
| Opti-MEM | Up to 20 μL | Up to 100 μL | Up to 500 μL |
