# Supplementary material for: Reprogrammed CRISPR-Cas13b suppresses SARS-CoV-2 replication and circumvents its mutational escape through mismatch tolerance
Source: Nat Commun. 2021 Jul 13;12:4270. doi: 10.1038/s41467-021-24577-9 (PMC8277810; doi:10.1038/s41467-021-24577-9)
Supplement: Supplementary file 11 — Description of Additional Supplementary Files [file 41467_2021_24577_MOESM11_ESM.pdf]

Title: Supplementary Data file 1.
Description: Single-nucleotide increment spacer sequence (DNA) of crRNAs covering the entire genome of SARS-CoV-2. The 30-nt spacer sequences were generated using an in-house script written in Python and are complementary to the target RNA. For each crRNA, we provide the matching location in the SARS-CoV-2 genome, the predicted secondary structure of the spacer and target sequences, the corresponding predicted minimum free energy (kcal/mol), and the name of the target on the SARS-CoV-2 genome as coding or intergenic regions. The alignment of crRNAs to the SARS-CoV-2 genome is based on a publicly available database on the UCSC genome browser. The data in this table are unfiltered and unranked.

Title: Supplementary Data file 2.
Description: Single-nucleotide increment spacer sequence of crRNAs covering the entire genome of SARS-CoV-2 are listed. The 30-nt spacer sequences are complementary to the target and were generated using in-house code written in Python. For each crRNA, we provide the matching coordinate in the SARS-CoV-2 genome, the predicted secondary structure of the spacer and target sequences, and the predicted minimum free energy (kcal/mol). The data in this table are filtered to remove spacer sequences that include 4 or more successive T residues that would prematurely terminate Pol III-driven spacer transcription and generate non-functional crRNAs. The crRNAs are ranked based on predicted secondary structures in the target and spacer sequence. The left table summarizes the number of crRNAs targeting each coding gene or non-coding intergenic regions in the SARS-CoV-2 genome.

Title: Supplementary Data file 3.
Description: Top scoring 838 crRNAs with a predicted open secondary structure in the spacer and target sequences. These crRNAs are designed to fully base-pair with various regions of the SRAS-CoV-2 genome and are predicted to achieve good silencing efficiency, given that the target accessibility governs target recognition processes in various RNA-guided eukaryotic and CRISPR effectors. crRNAs overlapping with mutation hotspots are highlighted in red, and it is recommended to avoid targeting these hotspot regions due to an increased risk of mutational escape compared to conserved genomic regions. The left table summarizes the number of crRNAs targeting each coding gene or non-coding intergenic regions in the SARS-CoV-2 genome. We found 827 crRNAs targeting coding regions and only 11 targeting intergenic regions that are not transcribed. Among the crRNAs targeting open reading frames, we found 578 targeting ORF1ab, 148 targeting the Spike (S), 25 targeting ORF3a, 4 targeting the envelope (E), 22 targeting M, 8 targeting ORF6, 4 targeting ORF7a, 30 targeting ORF7b, 4 targeting ORF8, and 4 targeting N.

Title: Supplementary Data file 4.
Description: 494 top scoring SARS-CoV-2 crRNAs after off-target prediction filtering. Stringent off-target prediction allowed the removal of all crRNAs with partial match (24-nt match or more) to human transcriptome that would generate 6- or fewer nucleotide mismatches at the spacer-target interface. crRNAs overlapping with mutation hotspots[1] are highlighted in red. The left table summarizes the number of crRNAs targeting each coding gene or non-coding intergenic regions in the SARS-CoV-2 genome.

Title: Supplementary Data file 5.
Description: Summary of transcriptome-wide off-target prediction applied to top-

scoring 838 crRNAs with various nucleotide mismatch cut-offs. No off-targeting crRNAs were detected with a full match, 1, or 2 nucleotide mismatches against the entire human transcriptome. However, 6, 21, 61, and 344 crRNAs among the 838 top scoring guides were predicted to have potential off-target activity with 3, 4, 5, and 6-nt mismatches with the target, respectively.

Title: Supplementary Data file 6.
Description: Genomic coordinates of various SARS-CoV-2 ORFs used to map crRNAs to their target locations on the SARS-CoV-2 genome in this bioinformatic study (adapted from UCSC genome browser).

Title: Supplementary Data file 7.
Description: Mutation hotspots in the SARS-CoV-2 genome are used to highlight crRNAs overlapping frequently mutated genomic regions. Adapted from Goswami et al1.

Title: Supplementary Data file 8.
Description: All experimentally tested crRNAs used in this study are listed in this table.

Title: Supplementary Data file 9.
Description: Transfection conditions of HEK 293T, VERO, and Calu-3 cell lines.
