# Supplementary material for: Reprogrammed CRISPR-Cas13b suppresses SARS-CoV-2 replication and circumvents its mutational escape through mismatch tolerance
Source: Nat Commun. 2021 Jul 13;12:4270. doi: 10.1038/s41467-021-24577-9 (PMC8277810; doi:10.1038/s41467-021-24577-9)
Supplement: Supplementary file 12 — Reporting Summary [file 41467_2021_24577_MOESM12_ESM.pdf]

nature research

Corresponding author(s): Mohamed Fareh
Sharon Lewin

Last updated by author(s): 17/06/21

Reporting Summary

Nature Research wishes to improve the reproducibility of the work that we publish. This form provides structure for consistency and transparency in reporting. For further information on Nature Research policies, see our Editorial Policies and the Editorial Policy Checklist.

Statistics

For all statistical analyses, confirm that the following items are present in the figure legend, table legend, main text, or Methods section.

n/a	Confirmed	
☐	☒	The exact sample size (n) for each experimental group/condition, given as a discrete number and unit of measurement
☐	☒	A statement on whether measurements were taken from distinct samples or whether the same sample was measured repeatedly
☐	☒	The statistical test(s) used AND whether they are one- or two-sided<br>*Only common tests should be described solely by name; describe more complex techniques in the Methods section.*
☒	☐	A description of all covariates tested
☒	☐	A description of any assumptions or corrections, such as tests of normality and adjustment for multiple comparisons
☐	☒	A full description of the statistical parameters including central tendency (e.g. means) or other basic estimates (e.g. regression coefficient) AND variation (e.g. standard deviation) or associated estimates of uncertainty (e.g. confidence intervals)
☐	☒	For null hypothesis testing, the test statistic (e.g. F, t, r) with confidence intervals, effect sizes, degrees of freedom and P value noted<br>*Give P values as exact values whenever suitable.*
☒	☐	For Bayesian analysis, information on the choice of priors and Markov chain Monte Carlo settings
☒	☐	For hierarchical and complex designs, identification of the appropriate level for tests and full reporting of outcomes
☒	☐	Estimates of effect sizes (e.g. Cohen's d, Pearson's r), indicating how they were calculated

Our web collection on statistics for biologists contains articles on many of the points above.

Software and code

Policy information about availability of computer code

Data collection	All data are available in the main text and suppl materials. All key plasmids generated in this study will be deposited in Addgene upon publication. The bioinformatic code for the design of single-nucleotide tiled crRNAs and spacer/target folding predictions are available here (https://github.com/data-vis/Covid19_crRNAs). All data generated by the bioinformatic codes are available in Suppl. tables 1-7.<br>Microscopy fluorescence images are processed and quantified using ImageJ (version 1.52a) software. Data analyses and visualizations (graphs) were performed in GraphPad Prism software version 7.<br>All flow cytometry profiles were analyzed using FlowJo V10 software (Tree Star Inc).
Data analysis	Single-nucleotide increment spacer sequence (DNA) of crRNAs covering the entire genome of SARS-CoV-2 were generated using inhouse script written in Python and are complementary to the target RNA. For each crRNA, we provide the matching location in the SARS-CoV-2 genome, the predicted secondary structure of the spacer and target sequences, and the corresponding predicted minimum free energy (kcal/mol). The data are analyzed and ranked in excel spreadsheets (Suppl. Tables 1-7). The predicted RNA secondary structures and minimum free energy were generated using the RNAfold program (ViennaRNA webservices; Lorenz, R. et al. ViennaRNA Package 2.0. Algorithms Mol. Biol. (2011) doi:10.1186/1748-7188-6-26.).

For manuscripts utilizing custom algorithms or software that are central to the research but not yet described in published literature, software must be made available to editors and reviewers. We strongly encourage code deposition in a community repository (e.g. GitHub). See the Nature Research guidelines for submitting code & software for further information.

Data

Policy information about availability of data

All manuscripts must include a data availability statement. This statement should provide the following information, where applicable:

- Accession codes, unique identifiers, or web links for publicly available datasets
- A list of figures that have associated raw data
- A description of any restrictions on data availability

All the raw data supporting the findings are available in the source Data file submitted with this manuscript.
All The SARS-CoV-2 virus isolates (ancestral, D614G, and B.1.1.7) are kind gift from Dr. Julian Druce (Victoria Infectious Disease Reference Lab, VIDRL) (Caly, L. et al. Isolation and rapid sharing of the 2019 novel coronavirus (SAR-CoV-2) from patients diagnosed with COVID-19 in Australia. Med. J. Aust. 212, (2020).
The unique accession codes for the ancestral and D614G SARS-CoV-2 strains used in this study are:
Ancestral SARS-CoV-2 = GISAID: EPI_ISL_406844; Genbank: MT007544.1
D614G SARS-CoV-2 = GISAID: EPI_ISL_419750; Genbank: MT450946.1
B1.1.7 SARS-CoV-2 = GISAID ID EPI_ISL_601443; GenBank: MW735442.1

Field-specific reporting

Please select the one below that is the best fit for your research. If you are not sure, read the appropriate sections before making your selection.

☒ Life sciences ☐ Behavioural & social sciences ☐ Ecological, evolutionary & environmental sciences

For a reference copy of the document with all sections, see nature.com/documents/nr-reporting-summary-flat.pdf

Life sciences study design

All studies must disclose on these points even when the disclosure is negative.

Sample size	The sample sizes were determined to match the standards in comparable studies available in the literature (Chunlong Xu et at, Nat Methods, 2021).
Data exclusions	Experiments were optimized in pilot assays before generating high-quality publication data. No data was excluded from the analysis.
Replication	All experiments in the virus-free models and infectious assays were repeated at least 3 times as biological replicates with the following exceptions:<br>- As mentioned in the figure legend, the data in Figure4 B-E were performed as biological replicate (N=2) due to large number of samples in this time course analysis and the restricted access to high confinement labs (PC3).<br>- As mentioned in the figure legend, Data in Fig 1G (screening 61 single-nucleotide resolution gRNAs targeting the Spike RNA) was performed once due to the large size of gRNAs screened and the large sequence overlap (redundancy) between two adjacent gRNAs (29 nt overlap out of 30nt spacer sequence).<br>After the initial optimization of the experimental conditions, all experiments were reproducible in independent experiments. RNA targeting with CRISPR-Cas13 is well-established and similar experiments have been reported by independent researchers.
Randomization	No randomization was used in this study. Due to the small sample randomization was not relevant for this study. Covariates were controlled for by running controls in parallel whenever is applicable. Appropriate controls (e.g. non targeting gRNAs, loading controls in WB, and time-course analysis) were used throughout the study.
Blinding	No blinding was used in this study. Blinding is not relevant to this study as RNA targeting with Cas13 is well-established in the field by independent groups using assays that do not require blinding (Chunlong Xu et at, Nat Methods, 2021). Most our experiments were performed, analysed, and confirmed by independent researchers in our labs. For infection assays, the VERO cells were transfected with Cas13 and various gRNAs at Petermac's PC2 labs and handed to researchers at the Peter Doherety Institute for SARS-CoV-2 infection and quantification of viral replication in an independent manner. The raw data were communicated to the involved authors for analysis and discussion.

Reporting for specific materials, systems and methods

We require information from authors about some types of materials, experimental systems and methods used in many studies. Here, indicate whether each material, system or method listed is relevant to your study. If you are not sure if a list item applies to your research, read the appropriate section before selecting a response.

Materials & experimental systems

n/a	Involved in the study
☐	☒ Antibodies
☐	☒ Eukaryotic cell lines
☒	☐ Palaeontology and archaeology
☒	☐ Animals and other organisms
☒	☐ Human research participants
☒	☐ Clinical data
☒	☐ Dual use research of concern

Methods

n/a	Involved in the study
☒	☐ ChIP-seq
☐	☒ Flow cytometry
☒	☐ MRI-based neuroimaging

Antibodies

Antibodies used	References and suppliers of the antibodies used in this study are listed in the manuscript together with a detailed western blot protocol.<br>- Anti-HA, mAb #2367, Cell Signaling Technology.<br>- Rabbit Anti-Mouse Immunoglobulins/HRP #p0260, Dako
Validation	We used commercial antibodies validated by the suppliers. We confirmed the validation as we used untrafected cells as control that did not show any band. Only cells transfected with constructs tagged with HA-tag showed a band at the expected molecular sizes.

Eukaryotic cell lines

Policy information about cell lines

Cell line source(s)	HEK 293T (ATCC CRL-3216); VERO (ATCC CCL-81); Calu-3 (ATCC HTB-55)
Authentication	Cell lines were authenticated by the supplier ATCC. We did not perform any additional authentication upon reception. We made a bulk stocks for each cell line after recovering from the original frozen vials. We discard the cells after ~20 passages, and thaw new cells from the liquid nitrogen stocks. Cell morphology was monitored at each passage by microscope.
Mycoplasma contamination	Cells were routinely tested (QPCR based test) and were mycoplasma negative.
Commonly misidentified lines (See ICLAC register)	No commonly misidentified cell lines were used in this manuscript.

Flow Cytometry

Plots

Confirm that:

☒ The axis labels state the marker and fluorochrome used (e.g. CD4-FITC).

☒ The axis scales are clearly visible. Include numbers along axes only for bottom left plot of group (a 'group' is an analysis of identical markers).

☒ All plots are contour plots with outliers or pseudocolor plots.

☒ A numerical value for number of cells or percentage (with statistics) is provided.

Methodology

Sample preparation	For monitoring cell transfection/transduction efficacy, cells were re-suspended in 200µL 1x PBS containing 2% FBS for flow cytometry analysis. All samples were analyzed by an LSR II (BD Biosciences), FORTESSA X20 (BD Biosciences) or FACSymphony (BD Biosciences). All flow cytometry profiles were analyzed using FlowJo V10 software (Tree Star Inc).
Instrument	All samples were analyzed by an LSR II (BD Biosciences), FORTESSA X20 (BD Biosciences) or FACSymphony (BD Biosciences)
Software	All flow cytometry profiles were analyzed using FlowJo V10 software (Tree Star Inc).
Cell population abundance	No sorting was used in this study.
Gating strategy	All gating strategies are shown in Suppl. Figure 3.VERO cells were transfected with Cas13-BFP, Spike-eGFP and various spike-targeting gRNA constructs prior to flow cytometry analysis. Cell aggregations were excluded by gating on single cells. Cells with fluorescence intensity higher than 10^3 were considered Cas13b-BFP positive and selected for further analysis. In the Cas13b-BFP positive subsets, the fluorescence intensity of Spike-eGFP is shown as dot plots and the silencing of various gRNAs was quantified using mean fluorescence intensity (MFI).

☒ Tick this box to confirm that a figure exemplifying the gating strategy is provided in the Supplementary Information.
